# Memory Functions in Obsessive–Compulsive Disorder

**DOI:** 10.3390/brainsci15050492

**Published:** 2025-05-07

**Authors:** Riccardo Gurrieri, Matteo Gambini, Elena Pescini, Diletta Mastrogiacomo, Gerardo Russomanno, Donatella Marazziti

**Affiliations:** Section of Psychiatry, Department of Clinical and Experimental Medicine, University of Pisa, Via Roma 57, 56100 Pisa, Italy; riccardogurrieri4@gmail.com (R.G.); gambinimatteo1996@gmail.com (M.G.); ele.pescini@gmail.com (E.P.); diletta.mastrogiacomo99@gmail.com (D.M.); g.russomanno93@gmail.com (G.R.)

**Keywords:** memory functions, obsessive–compulsive disorder, episodic memory, short-term memory, working memory, prospective memory, retrospective memory, memory distrust syndrome

## Abstract

**Background/Objectives:** Obsessive–compulsive disorder (OCD) is a complex psychiatric condition often associated with alterations in cognitive processes, including memory. Although memory dysfunction has been proposed as a contributing factor to the onset and maintenance of OCD symptoms, it remains debated whether these deficits reflect genuine cognitive impairments or maladaptive metacognitive processes, such as pathological doubt and memory distrust. This review aims to synthesize current findings on memory functioning in OCD, focusing on distinct memory systems and the role of metacognition. **Methods:** A comprehensive literature search was conducted across five databases (PubMed, Scopus, Embase, PsycINFO, and Google Scholar), covering studies up to April 2025. Search terms included “Obsessive-compulsive disorder”; “OCD”; “Memory dysfunction”; “Episodic memory”; “Working memory impairment”; “Prospective memory deficits”; “Checking compulsions”; “Memory confidence”; “Cognitive biases”. **Results:** Short-term memory appears generally preserved in OCD. Working memory deficits are consistently reported, especially in the visuospatial domain, and they are associated with difficulties in updating and clearing irrelevant information. Episodic memory impairments are common and often linked to inefficient encoding strategies and heightened cognitive self-consciousness. Prospective memory is frequently compromised under neutral conditions. Individuals with checking symptoms tend to show intact objective memory performance, despite reporting low memory confidence, supporting the concept of memory distrust. **Conclusions:** Memory dysfunction in OCD is multifaceted, involving both cognitive and metacognitive alterations. The evidence supports a model in which executive dysfunctions and memory-related beliefs contribute to compulsive behaviors more than objective memory failure. These insights highlight the need for integrative assessment protocols and personalized interventions targeting both cognitive performance and metacognitive appraisals.

## 1. Introduction

Memory is a fundamental cognitive function enabling individuals to encode, store, and retrieve information from past experiences, guiding decision-making and adaptive behavior in daily life [1]. The ability to remember past events is essential for learning, problem solving, and planning one’s own future. Beyond its role in healthy cognition, memory becomes particularly relevant in psychiatric conditions such as obsessive–compulsive disorder (OCD), where alterations, especially in memory confidence and metacognitive processes, may play a key role in the onset and maintenance of symptoms. This review specifically aims to synthesize current findings on memory functioning in OCD, examining both objective cognitive deficits and dysfunctional metacognitive beliefs.

Memory is not a unitary construct but a multifaceted one, and it can be distinguished into short-term memory (STM)—which holds limited information briefly (seconds), working memory (WM)—which allows its active manipulation, and long-term memory (LTM)—which enables the stable retention of information over time, each with distinct neurobiological underpinning [2,3,4]. LTM encompasses declarative (explicit) memory, which is responsible for consciously accessible knowledge such as facts and events, and non-declarative (implicit) memory, which involves unconscious skills and habitual responses [2].

The process of memory formation is typically divided into several stages: encoding, consolidation, storage, and retrieval. Encoding refers to the transformation of sensory inputs into neural codes called engrams, while consolidation stabilizes and strengthens them to become resistant to interference [5,6]. Storage refers to the maintenance of information over time, either in STM or LTM [5,7]. Finally, retrieval is the process of accessing stored information when needed [5,8].

Each of these stages is mediated by distinct neural networks, primarily involving the prefrontal cortex (PFC), hippocampus, and subcortical structures [2,9]. The PFC plays a crucial role in organizing and retrieving memories, as well as in WM processes and cognitive strategies enhancing learning [10]. The hippocampus is essential for the formation and consolidation of declarative memories, acting as a “bridge” between sensory information and its long-term storage in the cortical areas [2]. Meanwhile, subcortical structures, including the amygdala and basal ganglia, modulate emotional and motor responses related to memory, influencing consolidation based on emotional salience and repetition [11,12,13,14]. Long-term potentiation (LTP) is the mechanism underlying these processes, as a form of synaptic plasticity in which repeated stimulation strengthens neuronal connections, making synapses more susceptible to subsequent activation [15,16,17,18,19].

Memory is a dynamic and reconstructive process rather than a passive storage system: this means that retrieval is susceptible to distortions under the influence of internal cognitive factors and external environmental cues [20,21,22]. These processes are particularly relevant in psychiatric conditions, such as depression, anxiety, psychoses, OCD, and other, where disturbances in memory mechanisms can contribute to symptom severity and maladaptive behavioral patterns [23,24,25,26,27,28,29,30,31,32]. OCD is a psychiatric condition characterized by the presence of obsessions and/or compulsions, which cause significant distress and interfere with daily functioning according to the Diagnostic and Statistical Manual of Mental Disorders Fifth Edition Text Revision (DSM-5-TR) [33]; obsessions are defined as recurrent and persistent thoughts, urges, or images that are intrusive and cause marked anxiety or distress. These obsessions often involve themes such as contamination, symmetry, harm, or taboo thoughts, and individuals attempt to suppress or neutralize them through other thoughts or actions [34]. Compulsions, on the other hand, are repetitive behaviors or mental acts that an individual feels driven to perform in response to an obsession or according to rigidly applied rules. The purpose of compulsions is often to reduce anxiety or prevent a feared event, although these behaviors are not realistically connected to the feared consequences or are clearly excessive [35,36]. The distress and time-consuming nature of these compulsions significantly impair daily functioning, affecting interpersonal relationships, occupational performance, and overall quality of life [37].

Among OCD symptoms, checking behaviors are particularly relevant to memory functioning, as they often stem from pathological doubt, a persistent sense of uncertainty about one’s own actions or memories, often leading to compulsive behaviors despite intact performance [38].

As for the neurobiology, OCD is thought to be associated with alterations in the serotonin, dopamine, and glutamate systems [39], as well as hyperactivity in the cortico-striato-thalamo-cortical (CSTC) circuits, particularly in the orbitofrontal cortex (OFC), anterior cingulate cortex (ACC), and basal ganglia, which contributes to the persistence of intrusive thoughts and compulsive behaviors [40,41]. Dysfunction in these pathways would contribute to difficulties in inhibitory control, error monitoring, and cognitive flexibility, which may underlie repetitive behaviors and impairments in memory confidence [42].

Although some memory alterations have been reported in OCD, there is yet an ongoing debate focused on whether individuals with OCD exhibit true memory deficits, or if their difficulties are primarily due to pathological doubt and reduced memory confidence rather than impairments in encoding or retrieval [38,43,44,45]. This phenomenon, known as Memory Distrust Syndrome (MDS), suggests that individuals with OCD may doubt their own recollections, despite showing intact memory performance, leading to repetitive checking behaviors as a compensatory mechanism [46].

Studies on different types of memory processes in OCD have yielded mixed findings. Neuroimaging studies consistently demonstrated structural and functional abnormalities in fronto-striatal, limbic, and hippocampal circuits [40,47,48]. The hippocampus, a key structure in episodic memory (EM) and spatial navigation, has been found to exhibit reduced volume and altered connectivity with prefrontal and subcortical regions, potentially contributing to difficulties in memory retrieval [49,50,51]. Dysfunction in the ACC, which plays a role in error monitoring and conflict resolution, has been implicated in excessive doubt and compulsive checking behaviors, reinforcing maladaptive memory processing patterns [52,53], and the CSTC in cognitive flexibility and inhibitory control. These abnormalities might contribute to heightened uncertainty about past actions and compulsive checking behaviors [40,54,55].

Given the ongoing debate about whether memory dysfunctions in OCD stem from actual cognitive impairments or from maladaptive metacognitive beliefs such as pathological doubt and memory distrust, this review aims to clarify this distinction by synthesizing the current evidence on different memory systems in OCD and their role in symptom development, particularly in compulsive checking behaviors. Particular attention was devoted to examining how memory deficits and pathological doubt interact, shaping the clinical manifestations of OCD.

## 2. Materials and Methods

A comprehensive literature search was performed using the following electronic databases: PubMed, Scopus, Embase, PsycINFO, and Google Scholar. The search covered articles published up to April 2025 to ensure the inclusion of recent findings. The following combination of free-text terms was used: “Obsessive-compulsive disorder”; “OCD”; “Memory dysfunction”; “Episodic memory”; “Working memory impairment”; “Prospective memory deficits”; “Checking compulsions”; “Memory confidence”; “Cognitive biases”. Boolean operators (AND/OR) were applied to refine the search results. We manually screened reference lists from relevant articles to identify further studies that met our search criteria.

Given the heterogeneity of the methodologies used across studies, a qualitative synthesis was performed instead of a meta-analysis. Findings were grouped into thematic categories corresponding to different types of memory impairments and neurobiological underpinnings. The Discussion Section critically evaluates whether memory deficits in OCD reflect true impairments or are better explained by metacognitive dysfunctions such as pathological doubt and memory distrust.

## 3. Results

### 3.1. Short-Term Memory

The relationship between STM and OCD remains a controversial topic, with limited research and conflicting findings, partly because STM seems to be minimally impaired in OCD. A first study using the Rey–Osterrieth Complex Figure (ROCF) test to assess visuospatial memory showed that OCD patients performed poorly on both immediate and delayed recall while suggesting that deficits in OCD might be due to organizational difficulties rather than memory encoding per se [56]. Others reported that patients with OCD had slower categorization of words and poorer recall and recognition in word tasks, perhaps related to difficulties in using organizational strategies effectively under time pressure, which may disrupt memory encoding [57]. A subsequent research highlighted that STM remained intact in OCD patients, while executive functions were significantly impaired [58]. The main findings concerning the STM are shown in Table 1, which includes a selection of representative studies chosen for their methodological clarity and relevance to the topic.

In summary, research on STM impairments in OCD remains inconclusive, and further studies are needed to better understand the presence and nature of STM alterations in OCD and whether these deficits extend across all domains of STM or only affect specific components.

### 3.2. Working Memory

Working memory (WM) refers to the cognitive system responsible for temporarily holding and manipulating information during tasks such as learning, understanding, and reasoning. First introduced in the 1960s [65], the concept was later refined [4]. A model of WM was proposed consisting of two primary subsystems: the phonological loop, which processes verbal and phonetic information, and the visuospatial sketchpad, which processes visual and spatial information. A third component, the central executive system, regulates the previous two. Subsequently, the model was extended to include an episodic buffer, a component responsible for integrating information across modalities and linking to LTM [66].

Early studies suggested the presence of WM impairments in OCD, particularly of visuospatial WM (VSWM), as tasks become more complex [59,60,61]. Subsequent studies confirmed previous findings, while adding that memory deficits in OCD are not restricted to immediate VSWM memory but also involve extended LTM [67,68,69,70,71,72,73,74]. These impairments are thought to be related to deficits in executive function, particularly at the encoding stage [75], and might perhaps underpin the repetitive thoughts and behaviors typical of OCD [76].

In a study, the VSWM deficits observed in OCD patients were primarily related to the use of suboptimal executive strategies, rather than limitations in spatial storage capacity [62]. However, other scholars argued that WM deficits in OCD are relatively small and may not be clinically significant [77,78]. One possible explanation might be the reliance on declarative rather than procedural tasks to assess WM capacity. A distinction has been proposed between procedural and declarative WM processing, with declarative WM involving the storage of factual knowledge and procedural WM relating to rules for action [79]. Research suggests that declarative and procedural memory systems operate in parallel, implying separate WM subsystems [80,81,82]. It has been hypothesized that the small effect size for WM deficits in OCD is due to the predominance of declarative tasks in research assessments [83].

More recently, attempts have been made to elucidate the full extent of WM deficits in OCD. In one study carried out in 110 participants, the results showed that untreated OCD patients had significant deficits in all aspects of WM, including the phonological loop, visuospatial sketchpad, and central executive, supporting Baddeley’s original WM model [63]. Again, OCD patients performed worse in WM, cognitive flexibility, and response inhibition compared to patients with generalized anxiety disorder and social anxiety disorder. These findings suggest that OCD patients have significant cognitive impairments in WM that, however, did not differ significantly from those observed in the other two psychiatric groups [84].

Another study involving 90 OCD patients and 92 healthy controls assessed general memory and executive functions by means of the Wechsler Memory Scale-III (WMS-III), the Wisconsin Card Sorting Test (WCST), and the Stroop Color–Word Test (SCWT). Significant impairments were observed in the general memory and reaction time on the SCWT amongst the patients, together with impaired response inhibition. However, no intergroup difference was noted in set-shifting ability, suggesting that WM and other executive functions are particularly affected in OCD [64].

Not surprisingly, OCD patients had significantly higher levels of anxiety and poorer performance on VSWM tasks than controls. According to the authors, the negative correlation between VSWM and compulsive behaviors would indicate that impaired WM might exacerbate the relationship between anxiety and compulsive behaviors [85].

The possible relationship between WM deficits and repetitive thoughts in OCD patients represents a controversial topic. It has been suggested that these deficits are not due to an inability to suppress intrusive thoughts, but rather to difficulties in removing information from WM, leading to cognitive overload [86]. This theory warrants further investigation, and more research is needed to disentangle the role of WM in OCD.

The impact of gender differences in WM deficits is another factor that is often overlooked, as female patients with OCD tend to show more severe impairments in VSWM, particularly in tasks involving symmetry [77,87].

A few neuroimaging studies, including those using functional magnetic resonance imaging (fMRI), provided valuable insights into the neural substrates associated with WM dysfunction in OCD patients who performed significantly worse than controls on delayed recall tasks [88]. Increased activation was detected in regions such as the dorsolateral prefrontal cortex (DLPFC), left superior temporal gyrus (STG), left insula, and cuneus during a two-back task. In addition, OFC activity was positively correlated with symptom severity, suggesting its involvement in the pathophysiology of OCD [89].

Another group investigated the relationship between polygenic risk for OCD and WM performance by fMRI. Their findings showed reduced neural activity in the bilateral inferior parietal lobe and DLPFC, areas critical for WM processing. Interestingly, polygenic risk scores were found to be associated with neural activity in the OFC during WM tasks, suggesting a potential link between genetic vulnerability and altered brain functioning in OCD. These results support the concept of WM deficits as a “neurofunctional endophenotype” for OCD, reflecting both genetic and neurobiological vulnerability [89] (Table 1).

When comparing findings across studies, several patterns emerge. The VSWM deficits appear to be the most consistently reported impairment in OCD, especially under increased task complexity or time pressure [59,60,61,62,63]. In contrast, results regarding phonological loop or central executive function are more variable, with some studies reporting broad impairments [63], while others suggest only subtle or non-significant effects [77,78]. Differences in findings may be partly attributed to the type of task used (e.g., declarative vs. procedural WM assessments), with declarative tasks potentially underestimating deficits specific to OCD [79,83]. Moreover, clinical heterogeneity across samples, including symptom subtype, comorbidity, and medication status, likely contributes to inconsistent results. Notably, studies with larger and drug-naive samples tend to detect more widespread deficits [63,64], and neuroimaging data support the involvement of prefrontal regions (especially the DLPFC and OFC) in WM dysfunction in OCD, while OFC activity has been associated with genetic risk for OCD during WM processing [88,89]. Finally, a few studies indicate potential moderating effects of gender and anxiety, suggesting that WM impairments may be more pronounced in female patients or in the presence of high anxiety levels [77,85,87]. These findings provide important insights into the cognitive and neural mechanisms underlying OCD and may inform future interventions aimed at improving cognitive function in this population.

### 3.3. Long-Term Memory

#### Episodic Memory

Episodic memory (EM), an important component of LTM, allows people to recall and reconstruct personal experiences and to imagine future events. It was first introduced in 1972 to distinguish it from semantic memory [90]. In OCD, deficits in EM have been well documented and are considered an important aspect of the cognitive dysfunction of the disorder. A comparison of OCD patients with bipolar disorder (BD) and healthy controls showed that OCD patients had significant impairments in long-delayed free recall, with performance levels similar to those of BD patients, but worse than those of controls [75]. This deficit was specifically related to difficulties in using verbal organizational strategies during the learning phase, a pattern absent in bipolar patients. These problems of OCD patients with organization strategies, which are essential for efficient encoding and retrieval of information, have been confirmed in the same year [91]. A more extensive study comparing OCD patients with individuals with panic disorder and other anxiety disorders reported that OCD patients had the most pronounced EM impairments, along with deficits in verbal fluency, psychomotor speed, and executive functions [92].

The cognitive mechanisms underlying EM deficits in OCD have also been investigated by examining verbal EM in OCD patients who showed reduced immediate and delayed recall of complex verbal material, accompanied by increased self-reported cognitive self-consciousness. This cognitive self-consciousness was found to mediate the impairments in memory recall, suggesting that a thought-focused cognitive style might interfere with the encoding processes required for effective memory formation in OCD patients [93,94].

Gender differences in OCD have prompted further research into possible sex-related differences in EM. The results of a study carried out in 50 OCD patients (31 men, 19 women) and 50 matched controls noted significant impairments of EM strategies in male patients, as compared to male controls, while female patients did not. In addition, male OCD patients performed worse on organizational tasks than their female counterparts, suggesting that male patients may experience more severe cognitive dysfunction [95].

In addition, directed forgetting, a memory phenomenon in which individuals are instructed to forget certain information, has been used to investigate memory processes in OCD. Some data highlighted that OCD patients showed a reduced directed forgetting effect compared to controls. Although both groups recalled a similar number of words that they were instructed to forget, the OCD patients recalled significantly fewer words that they were instructed to retain. This finding suggests that OCD patients struggle with selective encoding and inhibition of irrelevant information, which may contribute to cognitive overload and memory dysfunction [96].

Neuroimaging data providing insight into the brain regions involved in EM deficits in OCD remain limited. To our knowledge, a single study conducted in pediatric patients reported hypoactivation in the dorsomedial prefrontal cortex (DMPFC) during verbal EM tasks [97]. Interestingly, the same study also identified increased activation in the posterior cingulate cortex (PCC) during memory retrieval, along with abnormal patterns in frontal and parietal regions. These alterations were associated with pathological doubt, suggesting that dysfunctional neural activity in these areas may contribute to EM impairments in OCD [97].

To sum up, EM deficits are an important feature of OCD, affecting free recall, organizational strategies, and selective encoding. These impairments are likely to be influenced by a thought-focused cognitive style that interferes with effective encoding.

Preliminary neuroimaging evidence from a single study suggests altered brain activity in regions such as the PFC and PCC, providing initial insight into the neural mechanisms underlying these cognitive impairments. In any case, there is an urgent need to deepen the neural and cognitive mechanisms behind EM dysfunction in OCD, which could help to develop targeted therapeutic interventions. Table 2 summarizes key studies on episodic memory performance in individuals with OCD. The studies were selected to reflect common methodological approaches and to illustrate consistent and divergent findings across samples.

### 3.4. Prospective and Retrospective Memory

Prospective memory (PM) refers to the cognitive processes involved in remembering to perform future actions, typically triggered by specific cues or intentions to be activated at a later time. This ability is crucial in everyday life and plays a major role in the successful performance of personal, social, and occupational activities [98]. Recognition involves the cognitive judgement of whether something has happened before, as being central to understanding how individuals manage their intentions and engage in future-oriented tasks [99]. By contrast, retrospective memory (RM) refers to the ability to recall information or events from the past, including facts, experiences, and previously formed intentions [100]. While closely related, PM and RM rely on partially distinct cognitive and neural mechanisms, with RM serving as a foundational component in the retrieval of stored intentions necessary for PM execution [101].

The PM has been extensively studied and found to be impaired in several neurological and psychiatric disorders. However, the study of PM function in OCD has only recently received attention.

To date, most studies of PM impairment in OCD have included participants with subclinical symptoms, and just a few studies have focused on individuals with full-blown OCD, but the available research has provided important insights into the underlying mechanisms of PM impairments in this population. In any case, when compared to other groups, subjects with OCD showed a significant PM deficit when exposed to neutral cues [102]. However, when the cues were negatively valenced or threat-related, they showed an improved PM response, suggesting that their attention to threat-related stimuli may help them overcome PM impairments associated with neutral cues. These findings are consistent with the notion that OCD patients may allocate attentional resources differently, prioritizing threat-related stimuli over neutral ones. A study explored PM functioning by assessing patients at three different time points: baseline, during the anticipation of a PM cue, and during the performance of a PM task [103]. The results showed that PM impairment in OCD was associated with excessive monitoring of external stimuli, which interfered with ongoing task performance and led to longer reaction times. It has been hypothesized that this might be due to hyperactivation of the thalamus and inhibition of the right DLPFC during the anticipation and execution of PM tasks, as noted in a Positron Emission Tomography (PET) study [103,104]. Another study specifically investigated time-based PM and found a significant association between PM performance, set shifting, and WM in 58 individuals with OCD and 58 matched controls [105]. A comprehensive cognitive battery was administered, including the modified WCST for cognitive flexibility, the Letter–Number Span Test for WM, and the Stroop Test for inhibitory control. Results indicated that while individuals with OCD were similar to controls on activity-based PM tasks, they showed a lower accuracy on time-based PM tasks and exhibited longer reaction times to event-based PM cues. These findings suggest that PM impairments in OCD may be cue-specific, with deficits in executive functions, particularly cognitive flexibility and WM updating, playing a critical role in time-based PM dysfunction [105].

A subsequent study expanded the investigation of PM impairments by comparing individuals with OCD, individuals with schizophrenia, and healthy controls, employing the Memory for Intentions Screening Test (MIST) to assess key cognitive domains including planning, mental flexibility, and cognitive inhibition [106]. Results revealed that patients with OCD exhibited significant deficits in both time-based and event-based PM tasks, with performance levels closely resembling those observed in individuals with schizophrenia [106].

More recently, a study [107] compared 30 individuals with OCD to 30 matched controls, employing a comprehensive set of assessment tools including the Dimensional Yale–Brown Obsessive–Compulsive Scale (Y-BOCS), the Hamilton Depression Rating Scale (HAMD), the General Health Questionnaire-12 (GHQ-12), and the Cambridge Prospective Memory Test (CAMPROMPT). The findings revealed a significant deficit in PM among OCD patients, specifically in event-based PM, while no significant differences emerged for time-based PM tasks. However, no significant differences were found in the severity or type of PM impairment, perhaps suggesting a transdiagnostic feature of PM dysfunction within OCD phenotypes.

While PM has been extensively investigated in OCD, data on RM remain limited. The available studies suggest that RM functioning is largely intact in OCD, which may explain the relatively limited focus on this domain within the literature [108]. Nevertheless, the current literature underscores the central role of PM in understanding the cognitive dysfunctions associated with OCD, with important implications for both diagnosis and the development of targeted therapeutic interventions. Table 3 summarizes a selection of representative studies on RM and PM in OCD. The included studies were chosen for their focus on real-time monitoring (e.g., event- and time-based PM), their methodological diversity, and their contribution to understanding compensatory mechanisms such as overmonitoring or altered self-reporting of symptoms. 

### 3.5. Compulsive Checking and Memory Distrust Syndrome

Compulsive checking represents the most common OCD subtype, characterized by persistent doubts and fears that lead to compulsions involving the repeated checking of activities, objects, instruments, appliances, and past actions [109]. Interestingly, checkers tend to show more pronounced memory impairments than other OCD subtypes [88,110]. Some data underline that these kinds of patients have slower reaction times on certain tasks, a phenomenon that is linked to pathological doubt that can interfere with memory processes. Indeed, checkers doubt their confidence in their own memories [111], especially when repeatedly exposed to threatening stimuli [38,112,113].

Further research described that compulsive checking in OCD is associated with reduced VSWM, with high checkers being less accurate than low checkers [85,113]. Furthermore, WM deficits have been hypothesized to play a role in compulsive checking behavior, although the relationship remains correlational and requires further investigation to establish causality [112,114]. Low WM scores predicted increased checking behaviors, while reinforcing the bidirectional relationship between memory deficits and compulsive checking [113].

Interestingly, patients with compulsive checking appear to have relatively intact EM, at variance with what is generally noted in OCD, but show significant deficits in PM, which may contribute to their compulsive behaviors [115,116]. In addition, checkers tend to exhibit more pronounced memory impairments than individuals with other OCD subtypes [88,110,115,116].

Patients with compulsive checking seem to have limited EM deficits and marked impairments of PM that would contribute to their compulsive behaviors [115,116]. These impairments are particularly relevant to self-directed action monitoring, as shown in frontal lobe tasks [117].

Despite these objective findings, individuals who engage in compulsive checking frequently hold the belief that their memory is unreliable, even when performance on memory tasks remains intact. Indeed, repetitive checking would increase familiarity with the items being checked by promoting conceptual processing, albeit inhibiting perceptual processing so that memories result in less vividness. This discrepancy between objective performance and subjective appraisal is the hallmark of Memory Distrust Syndrome (MDS), a term originally coined to describe the paradoxical phenomenon in which individuals distrust their memories despite showing no measurable memory deficits [46]. MDS has gained robust empirical support and is now considered a key metacognitive mechanism underlying compulsive checking in OCD [118,119]. In light of these findings, cognitive interventions that directly target dysfunctional memory beliefs have emerged as promising therapeutic tools. Specifically, cognitive–behavioral therapy (CBT) interventions focused on restructuring maladaptive metacognitive beliefs have been shown to reduce checking symptoms and enhance subjective memory confidence [120]. However, the improvement in perceived memory reliability has not been consistently linked to actual changes in memory performance, suggesting that targeting memory-related beliefs, rather than memory accuracy itself, may be a more effective treatment strategy. Nevertheless, additional longitudinal research is required to evaluate the long-term efficacy and durability of such approaches.

In conclusion, the literature underscores the complex and dynamic interplay between compulsive checking behaviors, memory performance, and metacognitive dysfunctions in OCD.

## 4. Discussion

The relationship between STM and OCD remains complex and controversial, with conflicting findings in the literature. While most recent studies have failed to identify significant differences between OCD patients and healthy controls, some older reports suggest subtle impairments. These inconsistencies may reflect methodological differences (e.g., task type, sample size), as well as clinical heterogeneity, such as symptom subtype or comorbidity, which can affect attentional resources and encoding strategies despite preserved memory capacity. Overall, STM, particularly in verbal and visuospatial modalities, appears relatively preserved compared to other memory systems [31,57,58]. However, impairments in executive functions such as planning and cognitive flexibility, commonly reported in OCD, may compromise the effective use of stored information [58]. According to the most robust hypothesis, STM dysfunction would not stem from a primary memory deficit, but rather from difficulties in employing organizational strategies, especially under time constraints or during complex tasks [31,57]. Therefore, memory in OCD may be structurally intact, albeit functionally impaired due to attentional biases and inefficient encoding processes [91].

Although some initial studies had suggested that WM deficits in OCD might be minimal [77], more recent and methodologically reliable investigations have consistently reported significant impairments, particularly in VSWM, with a notable gender difference showing greater impairments in female patients [59,60,61,63]. This gender difference may reflect sex-related variations in brain organization or symptom expression, such as a greater tendency toward symmetry-related compulsions in females, which could place additional demands on VSWM. These deficits are largely attributed to altered executive control, particularly the ability to update, manipulate, and clear information from WM. Rather than an inability to suppress intrusive thoughts, some authors propose that WM dysfunction in OCD is driven by difficulties in removing no-longer-relevant information with a consequent cognitive overload [63,84]. Neuroimaging studies reported altered activation in the DLPFC during WM tasks [88], and one study also found an association between OFC activity and genetic risk for OCD during WM processing [89], supporting the notion that executive dysfunctions may underlie WM impairments and be associated with compulsive behaviors.

EM deficits are a well-documented finding in OCD. Difficulties in both encoding and retrieval have been consistently observed, particularly in tasks requiring strategic organization and delayed recall [43,91,92]. These impairments have been linked to the inefficient use of encoding strategies and heightened cognitive self-consciousness—a metacognitive tendency to over-monitor one’s cognitive processes—which may interfere with effective memory consolidation [93]. In directed forgetting paradigms, OCD patients show a reduced ability to suppress irrelevant information, suggesting encoding difficulties that result in cognitive overload. Gender differences have also been reported, with male patients exhibiting greater deficits in episodic and organizational memory than their female counterparts [95]. Functional neuroimaging analyses, in this context, have highlighted a hypoactivation of the DMPFC and an increased activity in the PCC, which are brain regions implicated in memory retrieval and self-referential processing [97].

Given that many studies have been carried out in patients with subclinical forms of OCD, PM appears significantly impaired in OCD, particularly in the presence of neutral cues. Interestingly, performance seems to improve in response to negatively valenced or threat-related stimuli, possibly reflecting an attentional bias toward emotionally salient, threat-related content [102]. This supports the hypothesis that PM deficits may be associated with excessive external monitoring, which could interfere with internal task execution and may require emotionally intense stimuli to engage encoding processes effectively [103]. Impairments have been observed in both event-based and time-based PM tasks, although some findings on time-based PM remain inconsistent [105,106,107]. Several factors may account for these inconsistencies. Methodological heterogeneity across studies, such as differences in task structure, emotional salience, or ecological validity, can substantially influence PM performance. Sample-related variables, including symptom subtype, comorbidity, and medication status, may further modulate results. Moreover, the reliance of time-based PM on self-initiated retrieval and sustained attention may render it more vulnerable to individual differences in executive functioning. These elements should be considered in future research aiming to clarify the nature of PM impairments in OCD. In addition, neuroimaging data reported reduced activation of the DLPFC during both the anticipation and execution of PM tasks, reinforcing the role of executive dysfunction in PM impairments [104]. By contrast, RM appears largely preserved in OCD patients [108].

Patients with compulsive checking have been the most extensively studied in relation to memory functioning. These individuals experience pronounced pathological doubt concerning the reliability of their memories, leading to the phenomenon known as memory distrust. Despite demonstrating intact objective memory performance, they report lower memory confidence and reduced vividness of recollection [46,112,121]. A bidirectional relationship is proposed, wherein compulsive checking behavior may reinforce reduced memory confidence, which in turn could contribute to the maintenance of the compulsive cycle [115,116]. Cognitive–behavioral interventions targeting maladaptive memory beliefs have been shown to reduce checking behaviors, even without significant improvements in objective memory accuracy [120]. This suggests that therapeutic strategies should focus on metacognitive restructuring as a primary intervention target.

## 5. Conclusions

The present comprehensive review highlights the nuanced and multifactorial nature of memory dysfunction in OCD. While STM appears relatively spared in many cases, deficits in WM, EM, and PM are consistently observed, particularly in subtypes characterized by checking behaviors.

Rather than reflecting pure memory impairments, many of these deficits may be secondary to broader dysfunctions in executive processes, attentional control, and metacognitive beliefs. Neuroimaging studies support this view, revealing abnormal activation in key brain regions involved in memory and cognitive control, such as the dorsolateral prefrontal cortex, orbitofrontal cortex, and posterior cingulate cortex.

Importantly, individual factors such as gender, symptom subtype, and cognitive style appear to modulate the expression and severity of memory-related symptoms in OCD. This suggests a need for personalized cognitive and neuropsychological assessments in both research and clinical settings.

Future research should aim to disentangle true memory impairments from strategy-based or attention-related deficits and explore the interplay between memory confidence, intrusive thoughts, and compulsive behaviors. Longitudinal and intervention studies integrating neurocognitive and neurobiological data are essential to better understand the mechanisms driving cognitive dysfunction in OCD and to develop targeted treatments aimed at improving both memory functioning and clinical outcomes.

## Figures and Tables

**Table 1:** Main evidence on short-term and working memory (STM/WM) in patients with obsessive–compulsive disorder (OCD).

Authors	Type of Study	Task	Results
Shin et al., 2004 [56]	Case–control study; 30 OCD vs. 30 healthy controls	ROCF	Poor immediate and delayed recall in OCD patients
Sawamura et al., 2005 [57]	Case–control study; 16 OCD patients and 16 healthy controls	Word categorization, recall, and recognition	Slower categorization of words; poor recall/recognition under time pressure
Demeter et al., 2013 [58]	Case–control study; 30 OCD vs. 30 healthy controls	DST tasks, Corsi block- tapping task, and executive functions task	STM intact in OCD
Purcell et al., 1998 [59]	Case–control study; 23 OCD vs. 23 healthy controls	VSWM tasks	Impaired VSWM in OCD patients
Purcell et al., 1998 [60]	Comparative study; 30 OCD, 30 PD, 20 MDD, and 30 healthy controls	Computerized neuropsychological battery (spatial WM, recognition, attention, executive functions)	OCD impaired in spatial WM, recognition, and motor initiation
Van der Wee et al., 2003 [61]	fMRI study; 11 untreated female OCD vs. 11 matched controls	Parametric spatial n-back task	OCD patients impaired at high difficulty; hyperactivation in ACC
Perna et al., 2019 [62]	Case–control study; 30 OCD vs. 31 healthy controls	Spatial WM tasks	Impairments in spatial working memory in OCD patients
Yue et al., 2021 [63]	Case–control study; 55 drug-naive OCD patients vs. 55 healthy controls	DST, VSMT, and SCWT	Deficits in all WM components
Hamidian et al., 2022 [64]	Case–control study; 90 OCD vs. 92 healthy controls	WMS-III, WCST, and SCWT	Deficits in immediate, general, and working memory; WM scores predictive of OCD diagnosis

OCD: obsessive–compulsive disorder; PD: panic disorder; MDD: major depressive disorder; STM: short-term memory; WM: working memory; VSWM: visuospatial working memory; DST: digit span test; VSMT: visual space memory test; SCWT: Stroop Color–Word Test; WMS-III: Wechsler Memory Scale—Third Edition; WCST: Wisconsin Card Sorting Test; ROCF: Rey–Osterrieth Complex Figure; fMRI: functional magnetic resonance imaging; ACC: anterior cingulate cortex.

**Table 2:** Main evidence on long-term memory (LTM) in patients with obsessive–compulsive disorder (OCD).

Authors	Type of Study	Task	Results
Deckersbach et al., 2004 [75]	Comparative; 30 OCD vs. 30 BD-I vs. 30 healthy controls	CVLT	OCD impaired in long-delayed recall, mediated by poor use of semantic clustering strategies
Airaksinen et al., 2005 [92]	Population-based; 16 OCD vs. 175 healthy controls	RAVLT, WCST, FAS test, and TMT-A	OCD group showed impaired episodic memory
Exner et al., 2009 [93]	Case–control study; 23 OCD vs. 22 healthy controls	Story Recall Task, MCQ	Reduced recall mediated by cognitive self-consciousness; suggests disrupted encoding due to thought monitoring
Segalàs et al., 2010 [95]	Case–control study; 50 OCD vs. 50 healthy controls	ROCF, CVLT	Male OCD patients impaired in nonverbal memory; sex-specific differences in memory performance and strategy use
Konishi et al., 2011 [96]	Case–control study; 28 OCD vs. 17 healthy controls	Directed Forgetting Task	Reduced directed forgetting effect in OCD; deficits in selective encoding and retrieval inhibition

OCD: obsessive–compulsive disorder; BD-I: bipolar disorder type I; CVLT: California Verbal Learning Test; RAVLT: Rey Auditory Verbal Learning Test; WCST: Wisconsin Card Sorting Test; FAS test: Verbal Fluency Test (phonemic); TMT-A: Trail Making Test—Part A; ROCF: Rey–Osterrieth Complex Figure; MCQ: Metacognitions Questionnaire.

**Table 3:** Main evidence on retrospective and prospective memory (RM/PM) in patients with obsessive–compulsive disorder (OCD).

Authors	Type of Study	Task	Results
Marsh et al., 2009 [102]	Case–control study: 25 subclinical OCD vs. 50 matched controls	Event-based Prospective Memory Task	Deficit in neutral PM; enhanced with threat cues
Racsmány et al., 2011 [103]	Case–control study; 30 OCD vs. 30 healthy controls	Event-based PM task	OCD patients showed increased reaction time cost under PM load; supports overmonitoring hypothesis
Bhat et al., 2018 [106]	Case–control study; 22 OCD, 21 SCZ, 18 healthy controls	MIST (time- and event-based PM); WCST; Stroop; D-KEFS Tower	OCD impaired in both PM types; task substitution and omission errors prominent; only clock checking correlated with PM
Palit et al., 2022 [107]	Case–control study; 30 OCD vs. 30 healthy controls	CAMPROMPT (event- and time-based PM)	OCD group impaired in event-based PM; time-based PM intact; no differences across OCD subtypes
Gloster et al., 2008 [108]	Case reports study; 43 OCD patients	In vivo EMA vs. retrospective symptom recall and covariation estimation	Retrospective recall of OCD symptoms was accurate; overestimation of symptom covariation with external factors

OCD: obsessive–compulsive disorder; PM: prospective memory; RM: retrospective memory; MIST: Memory for Intentions Screening Test; WCST: Wisconsin Card Sorting Test; D-KEFS: Delis–Kaplan Executive Function System; CAMPROMPT: Cambridge Prospective Memory Test; EMA: Ecological Momentary Assessment; SCZ: schizophrenia.

## References

[1] Josselyn S.A., Tonegawa S. (2020). Memory engrams: Recalling the past and imagining the future. Science.

[2] Squire L.R., Genzel L., Wixted J.T., Morris R.G. (2015). Memory consolidation. Cold Spring Harb. Perspect. Biol..

[3] Atkinson R.C., Shiffrin R.M. (1968). Human memory: A proposed system and its control processes. Psychology of Learning and Motivation.

[4] Baddeley A.D., Hitch G., Bower G.H. (1974). Working memory. Psychology of Learning and Motivation.

[5] McDermott K.B., Roediger H.L. (2018). Memory (Encoding, storage, retrieval). General Psychology FA2018.

[6] Paller K.A., Wagner A.D. (2002). Observing the transformation of experience into memory. Trends Cogn. Sci..

[7] McGaugh J.L. (2000). Memory—A century of consolidation. Science.

[8] Squire L.R., Wixted J.T. (2011). The cognitive neuroscience of human memory since H.M. Annu. Rev. Neurosci..

[9] Eichenbaum H. (2017). Prefrontal–Hippocampal interactions in episodic memory. Nat. Rev. Neurosci..

[10] D’Esposito M., Postle B.R. (2015). The cognitive neuroscience of working memory. Annu. Rev. Psychol..

[11] Foerde K., Shohamy D. (2011). The role of the basal ganglia in learning and memory: Insight from Parkinson’s Disease. Neurobiol. Learn. Mem..

[12] Phelps E.A. (2004). Human emotion and memory: Interactions of the amygdala and hippocampal complex. Curr. Opin. Neurobiol..

[13] Kandel E.R., Pittenger C. (1999). The past, the future and the biology of memory storage. Philos. Trans. R. Soc. Lond. B.

[14] Kandel E.R., Klein M., Castellucci V.F., Schacher S., Goelet P. (1986). Some principles emerging from the study of short-and long-term memory. Neurosci. Res..

[15] Kandel E.R. (2001). The molecular biology of memory storage: A dialogue between genes and synapses. Science.

[16] Mayford M., Siegelbaum S.A., Kandel E.R. (2012). Synapses and memory storage. Cold Spring Harb. Perspect. Biol..

[17] Siegelbaum S.A., Kandel E.R. (1991). Learning-related synaptic plasticity: LTP and LTD. Curr. Opin. Neurobiol..

[18] Bliss T.V., Collingridge G.L. (1993). A Synaptic model of memory: Long-Term Potentiation in the hippocampus. Nature.

[19] Bliss T.V.P., Lømo T. (1973). Long-lasting potentiation of synaptic transmission in the dentate area of the anaesthetized rabbit following stimulation of the perforant path. J. Physiol..

[20] Koriat A., Goldsmith M., Pansky A. (2000). Toward a psychology of memory accuracy. Annu. Rev. Psychol..

[21] Schacter D.L., Coyle J.T. (1995). Memory Distortion: How Minds, Brains, and Societies Reconstruct the Past.

[22] Schwartz B.L. (2020). Memory: Foundations and Applications.

[23] Etkin A., Gyurak A., O’Hara R. (2013). A Neurobiological approach to the cognitive deficits of psychiatric disorders. Dialogues Clin. Neurosci..

[24] Goodman J., Marsh R., Peterson B.S., Packard M.G. (2014). Annual research review: The neurobehavioral development of multiple memory systems—Implications for childhood and adolescent psychiatric disorders. Child Psychol. Psychiatry.

[25] Muris P., Field A.P. (2008). Distorted cognition and pathological anxiety in children and adolescents. Cogn. Emot..

[26] Marazziti D., Consoli G., Picchetti M., Carlini M., Faravelli L. (2010). Cognitive impairment in major depression. Eur. J. Pharmacol..

[27] Rabner J.C., Ney J.S., Kendall P.C. (2024). Cognitive functioning in youth with anxiety disorders: A systematic review. Clin. Child. Fam. Psychol. Rev..

[28] Rock P.L., Roiser J.P., Riedel W.J., Blackwell A. (2014). Cognitive impairment in depression: A systematic review and meta-analysis. Psychol. Med..

[29] Chu R.S.T., Chu I.W.L., Yip E.W.-C., Chan J.K.N., Wong C.S.M., Hui C.L.-M., Chen E.Y.H., Chan S.K.W., Lee E.H.M., Lui S.S.Y. (2025). Cognitive functioning in people with psychotic experiences: A systematic review and meta-analysis study. Mol. Psychiatry.

[30] Benzina N., Mallet L., Burguière E., N’Diaye K., Pelissolo A. (2016). Cognitive dysfunction in Obsessive-Compulsive Disorder. Curr. Psychiatry Rep..

[31] Shin N.Y., Lee T.Y., Kim E., Kwon J.S. (2014). Cognitive functioning in Obsessive-Compulsive Disorder: A meta-analysis. Psychol. Med..

[32] Abramovitch A., Cooperman A. (2015). The cognitive neuropsychology of Obsessive-Compulsive Disorder: A critical review. J. Obs.-Compuls. Relat. Disord..

[33] APA—Diagnostic and Statistical Manual of Mental Disorders Fifth Edition Text Revision DSM-5-TR. https://www.appi.org/Products/DSM-Library/Diagnostic-and-Statistical-Manual-of-Mental-Di-.

[34] Abramowitz J.S., Taylor S., McKay D. (2009). Obsessive-Compulsive Disorder. Lancet.

[35] Starcevic V., Berle D., Brakoulias V., Sammut P., Moses K., Milicevic D., Hannan A. (2011). Functions of compulsions in Obsessive–Compulsive Disorder. Aust. N. Z. J. Psychiatry.

[36] Stein D.J., Costa D.L., Lochner C., Miguel E.C., Reddy Y.J., Shavitt R.G., van den Heuvel O.A., Simpson H.B. (2019). Obsessive–Compulsive Disorder. Nat. Rev. Dis. Primers.

[37] Eisen J.L., Mancebo M.A., Pinto A., Coles M.E., Pagano M.E., Stout R., Rasmussen S.A. (2006). Impact of Obsessive-Compulsive Disorder on quality of life. Compr. Psychiatry.

[38] Tolin D.F., Abramowitz J.S., Brigidi B.D., Amir N., Street G.P., Foa E.B. (2001). Memory and memory confidence in Obsessive–Compulsive Disorder. Behav. Res. Ther..

[39] Marazziti D., Albert U., Mucci F., Piccinni A. (2018). The glutamate and the immune systems: New targets for the pharmacological treatment of OCD. Curr. Med. Chem..

[40] Menzies L., Chamberlain S.R., Laird A.R., Thelen S.M., Sahakian B.J., Bullmore E.T. (2008). Integrating evidence from neuroimaging and neuropsychological studies of Obsessive-Compulsive Disorder: The orbitofronto-striatal model revisited. Neurosci. Biobehav. Rev..

[41] van den Heuvel O.A., van Wingen G., Soriano-Mas C., Alonso P., Chamberlain S.R., Nakamae T., Denys D., Goudriaan A.E., Veltman D.J. (2016). Brain circuitry of compulsivity. Eur. Neuropsychopharmacol..

[42] Doron G., Kyrios M. (2005). Obsessive Compulsive Disorder: A review of possible specific internal representations within a broader cognitive theory. Clin. Psychol. Rev..

[43] Deckersbach T., Otto M.W., Savage C.R., Baer L., Jenike M.A. (2000). The relationship between semantic organization and memory in Obsessive-Compulsive Disorder. Psychother. Psychosom..

[44] Hermans D., Engelen U., Grouwels L., Joos E., Lemmens J., Pieters G. (2008). Cognitive confidence in Obsessive-Compulsive Disorder: Distrusting perception, attention and memory. Behav. Res. Ther..

[45] Savage C.R., Deckersbach T., Wilhelm S., Rauch S.L., Baer L., Reid T., Jenike M.A. (2000). Strategic processing and episodic memory impairment in Obsessive Compulsive Disorder. Neuropsychology.

[46] van den Hout M., Kindt M. (2003). Phenomenological validity of an OCD-Memory model and the remember/know distinction. Behav. Res. Ther..

[47] Eng G.K., Sim K., Chen S.-H.A. (2015). Meta-analytic investigations of structural grey matter, executive domain-related functional activations, and white matter diffusivity in Obsessive Compulsive Disorder: An Integrative Review. Neurosci. Biobehav. Rev..

[48] Rotge J.-Y., Langbour N., Guehl D., Bioulac B., Jaafari N., Allard M., Aouizerate B., Burbaud P. (2010). Gray matter alterations in Obsessive–Compulsive Disorder: An anatomic likelihood estimation meta-analysis. Neuropsychopharmacology.

[49] De Wit S.J., Alonso P., Schweren L., Mataix-Cols D., Lochner C., Menchón J.M., Stein D.J., Fouche J.-P., Soriano-Mas C., Sato J.R. (2014). Multicenter voxel-based morphometry mega-analysis of structural brain scans in Obsessive-Compulsive Disorder. Am. J. Psychiatry.

[50] Kopřivová J., Congedo M., Horáček J., Praško J., Raszka M., Brunovskỳ M., Kohútová B., Höschl C. (2011). EEG source analysis in Obsessive–Compulsive Disorder. Clin. Neurophysiol..

[51] Reess T.J., Rus O.G., Gürsel D.A., Schmitz-Koep B., Wagner G., Berberich G., Koch K. (2018). Association between hippocampus volume and symptom profiles in Obsessive–Compulsive Disorder. NeuroImage Clin..

[52] Fitzgerald K.D., Welsh R.C., Gehring W.J., Abelson J.L., Himle J.A., Liberzon I., Taylor S.F. (2005). Error-related hyperactivity of the anterior cingulate cortex in Obsessive-Compulsive Disorder. Biol. Psychiatry.

[53] Ursu S., Stenger V.A., Shear M.K., Jones M.R., Carter C.S. (2003). Overactive action monitoring in Obsessive-Compulsive Disorder: Evidence from functional magnetic resonance imaging. Psychol. Sci..

[54] Pauls D.L., Abramovitch A., Rauch S.L., Geller D.A. (2014). Obsessive–Compulsive Disorder: An integrative genetic and neurobiological perspective. Nat. Rev. Neurosci..

[55] Saxena S., Rauch S.L. (2022). Functional neuroimaging and the neuroanatomy of Obsessive-Compulsive Disorder. Psychiatr. Clin. N. Am..

[56] Shin M.S., Park S.J., Kim M.S., Lee Y.H., Ha T.H., Kwon J.S. (2004). Deficits of organizational strategy and visual memory in Obsessive-Compulsive Disorder. Neuropsychology.

[57] Sawamura K., Nakashima Y., Inoue M., Kurita H. (2005). Short-term verbal memory deficits in patients with Obsessive-Compulsive Disorder. Psychiatry Clin. Neurosci..

[58] Demeter G., Racsmány M., Csigó K., Harsányi A., Németh A., Döme L. (2013). Intact short-term memory and impaired executive functions in Obsessive Compulsive Disorder. Ideggyógyászati Szle.

[59] Purcell R., Maruff P., Kyrios M., Pantelis C. (1998). Cognitive deficits in Obsessive–Compulsive Disorder on tests of frontal–striatal function. Biol. Psychiatry.

[60] Purcell R., Maruff P., Kyrios M., Pantelis C. (1998). Neuropsychological deficits in Obsessive-Compulsive Disorder: A comparison with unipolar depression, panic disorder, and normal controls. Arch. Gen. Psychiatry.

[61] van der Wee N.J., Ramsey N.F., Jansma J.M., Denys D.A., van Megen H.J., Westenberg H.M., Kahn R.S. (2003). Spatial working memory deficits in Obsessive Compulsive Disorder are associated with excessive engagement of the medial frontal cortex. Neuroimage.

[62] Perna G., Cavedini P., Riva A., Di Chiaro N.V., Bellotti M., Diaferia G., Caldirola D. (2019). The role of spatial store and executive strategy in spatial working memory: A comparison between patients with Obsessive–Compulsive Disorder and controls. Cogn. Neuropsychiatry.

[63] Yue J., Zhong S., Luo A., Lai S., He T., Luo Y., Wang Y., Zhang Y., Shen S., Huang H. (2021). Correlations between working memory impairment and neurometabolites of the prefrontal cortex in drug-naive Obsessive-Compulsive Disorder. Neuropsychiatr. Dis. Treat..

[64] Hamidian S., Pourshahbaz A., Ananloo E.S., Dolatshahi B., Ohadi M., Davoudi M. (2022). The story of memory and executive functions in Obsessive-Compulsive Disorder: A case-control study. Trends Psychiatry Psychother..

[65] Miller G.A., Eugene G., Pribram K.H. (1968). Plans and the structure of behaviour. Systems Research for Behavioral Science.

[66] Baddeley A. (2000). The Episodic Buffer: A new component of working memory?. Trends Cogn. Sci..

[67] Zielinski C.M. (1990). Neuropsychological Correlates in Obsessive-Compulsive Disorder.

[68] Zitterl W., Urban C., Linzmayer L., Aigner M., Demal U., Semler B., Zitterl-Eglseer K. (2001). Memory deficits in patients with DSM-IV Obsessive-Compulsive Disorder. Psychopathology.

[69] Woods C.M., Vevea J.L., Chambless D.L., Bayen U.J. (2002). Are compulsive checkers impaired in memory? A meta-analytic review. Clin. Psychol. Sci. Pract..

[70] Kuelz A.K., Hohagen F., Voderholzer U. (2004). Neuropsychological performance in Obsessive-Compulsive Disorder: A critical review. Biol. Psychol..

[71] Boldrini M., Del Pace L., Placidi G.P.A., Keilp J., Ellis S.P., Signori S., Placidi G.F., Cappa S.F. (2005). Selective cognitive deficits in Obsessive-compulsive Disorder compared to panic disorder with agoraphobia. Acta Psychiatr. Scand..

[72] Fontenelle L., Mendlowicz M., Mattos P., Versiani M. (2006). Neuropsychological findings in Obsessive-Compulsive Disorder and its potential implications for treatment. Curr. Psychiatry Rev..

[73] Morein-Zamir S., Craig K.J., Ersche K.D., Abbott S., Muller U., Fineberg N.A., Bullmore E.T., Sahakian B.J., Robbins T.W. (2010). Impaired visuospatial associative memory and attention in Obsessive Compulsive Disorder but no evidence for differential dopaminergic modulation. Psychopharmacology.

[74] Martinez-Gonzalez A.E., Piqueras-Rodriguez J.A. (2008). Neuropsychological update on Obsessive-Compulsive Disorder. Rev. Neurol..

[75] Deckersbach T., Savage C.R., Reilly-Harrington N., Clark L., Sachs G., Rauch S.L. (2004). Episodic memory impairment in Bipolar Disorder and Obsessive–Compulsive Disorder: The role of memory strategies. Bipolar Disord..

[76] Olley A., Malhi G., Sachdev P. (2007). Memory and executive functioning in Obsessive–Compulsive Disorder: A selective review. J. Affect. Disord..

[77] Abramovitch A., Abramowitz J.S., Mittelman A. (2013). The neuropsychology of adult Obsessive–Compulsive Disorder: A Meta-Analysis. Clin. Psychol. Rev..

[78] Ahmari S.E., Eich T., Cebenoyan D., Smith E.E., Blair Simpson H. (2014). Assessing neurocognitive function in psychiatric disorders: A roadmap for enhancing consensus. Neurobiol. Learn. Mem..

[79] Oberauer K. (2009). Chapter 2 Design for a working memory. Psychology of Learning and Motivation.

[80] Souza A.S., Oberauer K., Gade M., Druey M.D. (2012). Processing of representations in declarative and procedural working memory. Q. J. Exp. Psychol..

[81] Oberauer K. (2013). The focus of attention in working memory—From metaphors to mechanisms. Front. Hum. Neurosci..

[82] Barrouillet P., Camos V. (2014). Working Memory: Loss and Reconstruction.

[83] Shahar N., Teodorescu A.R., Anholt G.E., Karmon-Presser A., Meiran N. (2017). Examining procedural working memory processing in Obsessive-Compulsive Disorder. Psychiatry Res..

[84] Rosa-Alcázar Á., Parada-Navas J.L., García-Hernández M.D., Martínez-Murillo S., Olivares-Olivares P.J., Rosa-Alcázar A.I. (2021). Coping strategies, Anxiety and Depression in OCD and Schizophrenia: Changes during COVID-19. Brain Sci..

[85] Wang P., Yan Z., Chen T., Cao W., Yang X., Meng F., Liu Y., Li Z. (2023). Visuospatial working memory capacity moderates the relationship between Anxiety and OCD related checking behaviors. Front. Psychiatry.

[86] Boo Y.J., Kim D.-W., Park J.Y., Kim B.S., Chang J.W., Kang J.I., Kim S.J. (2023). Altered prefrontal beta oscillatory activity during removal of information from working memory in Obsessive-Compulsive Disorder. BMC Psychiatry.

[87] Martoni R.M., Salgari G., Galimberti E., Cavallini M.C., O’Neill J. (2015). Effects of gender and executive function on visuospatial working memory in adult Obsessive–Compulsive Disorder. Eur. Arch. Psychiatry Clin. Neurosci..

[88] Nakao T., Nakagawa A., Nakatani E., Nabeyama M., Sanematsu H., Yoshiura T., Togao O., Tomita M., Masuda Y., Yoshioka K. (2009). Working memory dysfunction in Obsessive–Compulsive Disorder: A neuropsychological and Functional MRI study. J. Psychiatr. Res..

[89] Heinzel S., Kaufmann C., Grützmann R., Klawohn J., Riesel A., Bey K., Heilmann-Heimbach S., Weinhold L., Ramirez A., Wagner M. (2021). Polygenic risk for Obsessive-Compulsive Disorder (OCD) predicts brain response during working memory task in OCD, unaffected relatives, and healthy controls. Sci. Rep..

[90] Tulving E. (1972). Episodic and semantic memory. Organ. Mem..

[91] Roth R.M., Baribeau J., Milovan D.L., O’Connor K. (2004). Speed and accuracy on tests of executive function in Obsessive–Compulsive Disorder. Brain Cogn..

[92] Airaksinen E., Larsson M., Forsell Y. (2005). Neuropsychological functions in Anxiety Disorders in population-based samples: Evidence of episodic memory dysfunction. J. Psychiatr. Res..

[93] Exner C., Kohl A., Zaudig M., Langs G., Lincoln T.M., Rief W. (2009). Metacognition and episodic memory in Obsessive-Compulsive Disorder. J. Anxiety Disord..

[94] Exner C., Martin V., Rief W. (2009). Self-focused ruminations and memory deficits in Obsessive–Compulsive Disorder. Cogn. Ther. Res..

[95] Segalàs C., Alonso P., Labad J., Real E., Pertusa A., Jaurrieta N., Jiménez-Murcia S., Menchón J.M., Vallejo J. (2010). A Case-control study of sex differences in strategic processing and episodic memory in Obsessive-Compulsive Disorder. Compr. Psychiatry.

[96] Konishi M., Shishikura K., Nakaaki S., Komatsu S.-I., Mimura M. (2011). Remembering and forgetting: Directed forgetting effect in Obsessive-Compulsive Disorder. Neuropsychiatr. Dis. Treat..

[97] Batistuzzo M.C., Balardin J.B., da Graça Morais Martin M., Hoexter M.Q., Bernardes E.T., Borcato S., de Marco E Souza M., Querido C.N., Morais R.M., De Alvarenga P.G. (2015). Reduced prefrontal activation in pediatric patients with Obsessive-Compulsive Disorder during verbal episodic memory encoding. J. Am. Acad. Child Adolesc. Psychiatry.

[98] Meacham J.A., Singer J. (1977). Incentive effects in prospective remembering. J. Psychol..

[99] Mandler G. (1980). Recognizing: The judgment of previous occurrence. Psychol. Rev..

[100] Roediger H.L., Zaromb F.M., Goode M.K., Byrne J.H. (2008). 1.02—A typology of memory terms. Learning and Memory: A Comprehensive Reference.

[101] Burgess P.W., Shallice T. (1997). The relationship between prospective and retrospective memory: Neuropsychological evidence. Cognitive Models of Memory.

[102] Marsh R.L., Brewer G.A., Jameson J.P., Cook G.I., Amir N., Hicks J.L. (2009). Threat-related processing supports prospective memory retrieval for people with obsessive tendencies. Memory.

[103] Racsmány M., Demeter G., Csigó K., Harsányi A., Németh A. (2011). An experimental study of prospective memory in Obsessive-Compulsive Disorder. J. Clin. Exp. Neuropsychol..

[104] Burgess P.W., Quayle A., Frith C.D. (2001). Brain regions involved in prospective memory as determined by Positron Emission Tomography. Neuropsychologia.

[105] Yang T., Peng Z., Wang Y., Geng F., Miao G., Shum D.H., Cheung E.F., Chan R.C. (2015). The Nature of prospective memory deficit in patients with Obsessive–Compulsive Disorder. Psychiatry Res..

[106] Bhat N.A., Sharma V., Kumar D. (2018). Prospective memory in Obsessive Compulsive Disorder. Psychiatry Res..

[107] Palit A., Roy P.K., Saha P.K. (2022). Role of prospective memory in Obsessive Compulsive Disorder. Indian J. Psychol. Med..

[108] Gloster A.T., Richard D.C., Himle J., Koch E., Anson H., Lokers L., Thornton J. (2008). Accuracy of retrospective memory and covariation estimation in patients with Obsessive–Compulsive Disorder. Behav. Res. Ther..

[109] Strauss A.Y., Fradkin I., McNally R.J., Linkovski O., Anholt G.E., Huppert J.D. (2020). Why check? A meta-analysis of checking in Obsessive-Compulsive Disorder: Threat vs. Distrust of Senses. Clin. Psychol. Rev..

[110] Yazarloo M., Sarafraz M.R., Jabbari S., Gholipour T., Hashemi T. (2024). Comparison of retrospective and prospective memory in subtypes of Obsessive-Compulsive Disorder. Eur. J. Transl. Myol..

[111] MacDonald P.A., Antony M.M., Macleod C.M., Richter M.A. (1997). Memory and confidence in memory judgements among individuals with Obsessive Compulsive Disorder and non-clinical controls. Behav. Res. Ther..

[112] Harkin B., Kessler K. (2009). How Checking breeds doubt: Reduced performance in a simple working memory task. Behav. Res. Ther..

[113] Jaafari N., Frasca M., Rigalleau F., Rachid F., Gil R., Olié J.-P., Guehl D., Burbaud P., Aouizerate B., Rotgé J.-Y. (2013). Forgetting what you have checked: A link between working memory impairment and checking behaviors in Obsessive-Compulsive Disorder. Eur. Psychiatry.

[114] Harkin B., Kessler K. (2011). The role of working memory in compulsive checking and OCD: A systematic classification of 58 experimental findings. Clin. Psychol. Rev..

[115] Cuttler C., Graf P. (2009). Checking-in on the memory deficit and meta-memory deficit theories of compulsive checking. Clin. Psychol. Rev..

[116] Cuttler C., Graf P. (2007). Sub-clinical compulsive checkers’ prospective memory is impaired. J. Anxiety Disord..

[117] Roth R.M., Baribeau J. (1996). Performance of subclinical compulsive checkers on putative tests of frontal and temporal lobe memory functions. J. Nerv. Ment. Dis..

[118] Radomsky A.S., Dugas M.J., Alcolado G.M., Lavoie S.L. (2014). When more is less: Doubt, Repetition, memory, metamemory, and compulsive checking in OCD. Behav. Res. Ther..

[119] Boschen M.J., Vuksanovic D. (2007). Deteriorating memory confidence, responsibility perceptions and repeated checking: Comparisons in OCD and control samples. Behav. Res. Ther..

[120] Alcolado G.M., Radomsky A.S. (2016). A novel cognitive intervention for compulsive checking: Targeting maladaptive beliefs about memory. J. Behav. Ther. Exp. Psychiatry.

[121] Radomsky A.S., Gilchrist P.T., Dussault D. (2006). Repeated checking really does cause memory distrust. Behav. Res. Ther..

